# High Fat Diet and Inflammation – Modulation of Haptoglobin Level in Rat Brain

**DOI:** 10.3389/fncel.2015.00479

**Published:** 2015-12-15

**Authors:** Maria Stefania Spagnuolo, Maria Pina Mollica, Bernardetta Maresca, Gina Cavaliere, Carolina Cefaliello, Giovanna Trinchese, Rosaria Scudiero, Marianna Crispino, Luisa Cigliano

**Affiliations:** ^1^Laboratory of Animal Physiology, Department of Bio-Agrofood Science, Institute for Animal Production System in Mediterranean Environment, National Research CouncilNaples, Italy; ^2^Department of Biology, University of Naples Federico IINaples, Italy

**Keywords:** haptoglobin, hemoglobin, TNF-alpha, nitro-tyrosine, high-fat diet, human astrocytoma cell line U-87 MG, rat, hippocampus

## Abstract

Obesity and dietary fats are well known risk factors for the pathogenesis of neurodegenerative diseases. The analysis of specific markers, whose brain level can be affected by diet, might contribute to unveil the intersection between inflammation/obesity and neurodegeneration. Haptoglobin (Hpt) is an acute phase protein, which acts as antioxidant by binding free haemoglobin (Hb), thus neutralizing its pro-oxidative action. We previously demonstrated that Hpt plays critical functions in brain, modulating cholesterol trafficking in neuroblastoma cell lines, beta-amyloid (Aβ) uptake by astrocyte, and limiting Aβ toxicity on these cells. A major aim of this study was to evaluate whether a long term (12 or 24 weeks) high-fat diet (HFD) influences Hpt and Hb expression in rat hippocampus. We also assessed the development of obesity-induced inflammation by measuring hippocampal level of TNF-alpha, and the extent of protein oxidation by titrating nitro-tyrosine (N-Tyr). Hpt concentration was lower (*p* < 0.001) in hippocampus of HFD rats than in control animals, both in the 12 and in the 24 weeks fed groups. HFD was also associated in hippocampus with the increase of Hb level (*p* < 0.01), inflammation and protein oxidative modification, as evidenced by the increase in the concentration of TNF-alpha and nitro-tyrosine. In fact, TNF-alpha concentration was higher in rats receiving HFD for 12 (*p* < 0.01) or 24 weeks (*p* < 0.001) compared to those receiving the control diet. N-Tyr concentration was more elevated in hippocampus of HFD than in control rats in both 12 weeks (*p* = 0.04) and 24 weeks groups (*p* = 0.01), and a positive correlation between Hb and N-Tyr concentration was found in each group. Finally, we found that the treatment of the human glioblastoma-astrocytoma cell line U-87 MG with cholesterol and fatty acids, such as palmitic and linoleic acid, significantly impairs (*p* < 0.001) Hpt secretion in the extracellular compartment. We hypothesize that the HFD-dependent decrease of Hpt in hippocampus, as associated with Hb increase, might enhance the oxidative stress induced by free Hb. Altogether our data, identifying Hpt as a molecule modulated in the brain by dietary fats, may represent one of the first steps in the comprehension of the molecular mechanisms underlying the diet-related effects in the nervous system.

## Introduction

Obesity relationship with various health problems has been well described in several recent studies (Haslam and James, 2005; Malnick and Knobler, 2006). In the last decade, obesity, and dietary fats were identified as risk factors for cognitive decline and various types of neurodegenerative dementias (Kalmijn, 2000; Solfrizzi et al., 2003; Gustafson, 2008; Craft, 2009; Sellbom and Gunstad, 2012). Indeed, human epidemiological studies revealed a correlation between a high-fat diet (HFD) and cognitive impairment (Elias et al., 2003; Morris et al., 2004; Zhang et al., 2006), as well as between body mass index and Alzheimer’s Disease (AD) development (Benito-León et al., 2013; Reinert et al., 2013). In particular, it was demonstrated that a diet enriched with saturated and trans fatty acids is associated with increased risk for AD (Kalmijn et al., 1997; Luchsinger et al., 2002; Morris et al., 2003). Biological mechanisms underlying damaging effects of obesity on brain are still poorly understood, although studies on experimental models and humans have shown that obesity is associated with increased brain oxidative stress (Mattson et al., 2003; Zhang et al., 2005; Souza et al., 2007) and neuroinflammation (Thirumangalakudi et al., 2008; Pistell et al., 2010; Freeman et al., 2014), both implicated in the pathogenesis of neurodegenerative diseases (Freeman et al., 2014). Therefore, the identification of molecules that can be modulated by dietary fats, might contribute to clarify the specific obesity-related effects on brain physiology and pathology.

In this frame, we demonstrated that Haptoglobin (Hpt), an acute-phase protein of inflammation, binds Apolipoprotein E (ApoE), influencing ApoE-mediated cholesterol trafficking in blood (Cigliano et al., 2009) and in neuroblastoma cell lines (Spagnuolo et al., 2014a). Further, we have recently reported that Hpt also affects ApoE-dependent 24(S)-hydroxycholesterol esterification (La Marca et al., 2014), a key step for cholesterol removal from the brain.

Hpt is synthesized primarily by hepatocytes, and in white adipose tissue (WAT; Friedrichs et al., 1995; Quaye, 2008), and its blood levels vary according to the volume of adiposity in the body (Chiellini et al., 2004). Hpt is so far known for its ability to bind free Haemoglobin (Hb), to transport it to the liver and therefore to limit the pro-oxidative action of free Hb (Quaye, 2008). Also, we previously demonstrated that Hpt acts as antioxidant, by preventing Apolipoprotein A-I (ApoA-I) and ApoE oxidation by free radical species (Salvatore et al., 2007, 2009). Although the presence of Hpt in the brain was firstly suggested as a marker of blood-brain barrier (BBB) dysfunction (Chamoun et al., 2001), different studies demonstrated that Hpt is produced in this compartment, in response to stress stimuli (Lee et al., 2002; Borsody et al., 2006; Zhao et al., 2009), as well as by human astrocytes *in vitro* (Maresca et al., 2015). Further, increased Hpt concentration was found in cerebrospinal fluid (CSF) from AD patients (Johnson et al., 1992; Yerbury and Wilson, 2010), and increased oxidation of this protein was described in plasma of AD patients (Cocciolo et al., 2012). In addition, we recently demonstrated that Hpt increases with the age in rat hippocampus as well as in human CSF (Spagnuolo et al., 2014a).

Beyond its Hb-binding function, Hpt also acts as extracellular chaperone, by inhibiting amyloid beta (Aβ) fibril formation *in vitro* (Wilson et al., 2008; Yerbury et al., 2009), colocalizes with amyloid plaques in AD (Powers et al., 1981; Veerhuis et al., 2003; Yerbury et al., 2005), and was proposed as potential biomarker of AD (Thambisetty, 2010). In addition, we recently provided evidence that Hpt, due to its ability to bind both ApoE and Aβ, promotes the formation of the ApoE/Aβ complex (Spagnuolo et al., 2014b), which is crucial to prevent or limit Aβ neurotoxicity and to promote its clearance. Hpt modulation of Aβ uptake by astrocyte and its activity of limiting Aβ toxicity in these cells was also reported (Maresca et al., 2015).

As dietary composition can negatively affect hippocampal function (Hsu and Kanoski, 2014), and Hpt modulates ApoE-dependent cholesterol homeostasis in the brain (Spagnuolo et al., 2014a), a major aim of this study was to evaluate whether a long term high fat diet influences Hpt expression in rat hippocampus. This brain region, crucial for learning and memory, is considered one of the most vulnerable sites in early AD and other neurodegenerative diseases development (Gómez-Isla et al., 1996; Price et al., 2001), and is particularly susceptible to disruption by dietary factors. In addition, in view of the previously described Hpt antioxidant function (Salvatore et al., 2007, 2009; Quaye, 2008), we also investigated whether diet-dependent Hpt changes are associated with inflammation and with changes in the level of nitro-tyrosine (N-Tyr), here selected as marker of the extent of protein oxidative modification.

## Materials and Methods

### Materials

Bovine serum albumin fraction V (BSA), human Hb, phosphatidylcholine (PC) from egg yolk, 2-linoleoyl-1-palmitoyl-sn-glycero-3-phosphocholine (LP), rabbit anti-human Hpt IgG, mouse anti-β actin IgG, goat anti-rabbit Horseradish Peroxidase-conjugated IgG (GAR-HRP), goat anti-mouse Horseradish Peroxidase-conjugated IgG (GAM-HRP), rabbit anti-human Hb, mouse anti-human Hpt, and MTT [3-(4,5-dimethylthiazol-2-yl)-2,5-diphenyltetrazolium bromide], were purchased from Sigma–Aldrich (St. Louis, MO, USA). Rabbit anti-N-Tyr of Covalab was purchased by Vincibiochem (Vinci, Italy). Rabbit anti-rat Hpt was from ICL Lab (distributed by Prodotti Gianni, Milano, Italy). The dye reagent for protein titration, enhanced chemiluminescence (ECL) reagents, and the polyvinylidene difluoride (PVDF) membrane were from Bio-Rad (Bio-Rad, Hercules, CA, USA). Polystyrene 96-wells ELISA MaxiSorp plates, with high affinity to proteins with mixed hydrophilic/hydrophobic domains, were purchased from Nunc (Roskilde, Denmark). Kodak Biomax light film, Sephacryl S-200, CNBr-activated Sepharose 4 Fast Flow and Blue Sepharose 6 Fast Flow resins were from GE-Healthcare Life Sciences (Milano, Italy). DMEM, fetal bovine serum (FBS), L-glutamine, penicillin and streptomycin were from Gibco (Life Technologies Italy, Monza, Italy). Cell culture flasks (25 and 75 cm^2^), 6 and 96-well cell culture plates, and sterile pipettes of Beckton-Dickinson (Milan, Italy) were used.

### Animal and Experimental Design

Two months old male Wistar rats (Charles River, Calco, Como, Italy), with similar body weight (340 ± 10 g), were individually caged in a temperature-controlled room (23 ± 1°C) with a 12-h light/12-h dark cycle. Animals were housed in the Animal Care Facility at the Department of Biology, with water *ad libitum*, and they were randomly divided into four experimental groups. Two groups (control) received a standard diet (control diet, CD; 10.6% fat J/J; Mucedola 4RF21; Settimo Milanese, Milan, Italy) for 12 (*N* = 8; CD 12) or 24 (*N* = 7; CD 24) weeks. The other two groups (high-fat) received a HFD rich in lard (40% fat J/J) for 12 (*N* = 6; HFD 12) or 24 (*N* = 7; HFD 24) weeks. The composition of all dietary regimens is reported in **Table 1.** During the treatments, the body weight and food intake were monitored daily to calculate weight gain and gross energy intake. Spilled food were collected daily for precise food intake calculation.

**Table 1:** Diet composition.

	Control diet	High lard diet (g/100g diet)
Standard feed	100	51.3
Casein^a^ g	-	9.25
Lard g	-	21.8
Sunflower oil g	-	1.24
AIN 76 mineral mix^b^ g	-	1.46
AIN 76 Vitamin mix^c^ g	-	0.42
Choline bitartrato	-	0.08
Methionine g	-	0.12
Energy density kJ/g diet	15.88	20.00
Protein %	29	29
Lipid %	10.6	40
Carbohydrate %	60.4	31


*^a^Purified high-nitrogen casein containing 88% protein, ^b^American Institute of Nutrition (1977), ^c^American Institute of Nutrition (1980).*

At the end of the experimental period, animals of each group were anesthetized by chloral hydrate (40 mg/100 g body wt) and killed by decapitation. Blood was collected and serum was obtained by centrifuging at 1000 × *g* for 10 min before storing at -80°C until the biochemical measurements.

The brains were quickly removed and hippocampus was dissected on ice. Samples were snap frozen in liquid nitrogen immediately and stored at -80°C for subsequent RNA and protein isolation.

The evaluation of fat and protein content in animal carcasses was conducted according to a published protocol (Iossa et al., 2002).

This study was carried out in strict accordance with the Institutional Guidelines and complied with the Italian D.L. no.116 of January 27, 1992 of Ministero della Salute and associated guidelines in the European Communities Council Directive of November 24, 1986 (86/609/ECC). All animal procedures reported herein were approved by the Institutional Animal Care and Use Committee (CSV) of University of Naples Federico II. All efforts were made to minimize animal suffering and to reduce the number of animals used.

### Cell Culture

The human glioblastoma-astrocytoma cell line U-87 MG was kindly provided by the Institute of Genetics and Biophysics (CNR, Naples, Italy). Cells (500,000) were seeded in 50 ml tissue culture flasks (25 cm^2^ surface), and grown in DMEM supplemented with 10% FBS, 2 mM L-glutamine, 100 U/ml penicillin, and 100 μg/ml streptomycin (complete medium) at 37°C under humidified atmosphere of 5% CO_2_ in air. The medium was changed twice a week, and cells were sub-cultivated when confluent.

U-87 MG cells were seeded into 6-well plates (at 400,000 cells/well density) in complete medium, and incubated (37°C, 5% CO_2_) for 20 h. After removal of the medium, and washing with DMEM, the cells were used in the assays described below.

### Titration of Serum Hpt

Haptoglobin concentration in individual samples was measured by ELISA. Samples were diluted (1:1000, 1:3000, 1:9000, 1:20000, and 1:40000) with coating buffer (7 mM Na_2_CO_3_, 17 mM NaHCO_3_, 1.5 mM NaN_3_, pH 9.6), and aliquots (50 μl) were then incubated in the wells of a microtiter plate (Immuno MaxiSorp; overnight, 4°C). After four washes by T-TBS (130 mM NaCl, 20 mM Tris-HCl, 0.05% Tween 20, pH 7.4) and four washes by high salt TBS (500 mM NaCl in 20 mM Tris-HCl at pH 7.4), the wells were blocked with TBS containing 0.5% BSA (1 h, 37°C). After washing, the wells were incubated (1 h, 37°C) with 60 μl of rabbit anti-rat IgG (1: 500 dilution in T-TBS containing 0.25% BSA) followed by 60 μl of GAR-HRP IgG (1:3000 dilution). Peroxidase-catalyzed color development from *o*-phenylenediamine was measured at 492 nm. The calibration curve was obtained by assaying the immunoreactivity of 2, 1, 0.5, 0.25, 0.1, 0.05, 0.03 ng of purified Hpt 1-1.

### Analysis of TNF-Alpha Concentration in Hippocampus

For quantification of TNF-alpha, proteins were extracted from slices hippocampus by homogenizing frozen tissues in lysis buffer (100 mM Tris/HCl, pH 7.0, 1 M NaCl, 4 mM EDTA.Na_2_, 2% Triton X-100, 0.1% sodium azide) containing Tissue Protease Inhibitor Cocktail (Sigma–Aldrich, 1:1000, v/v). Hippocampal homogenates were centrifuged at 14 000 × *g* for 30 min at 4°C and soluble samples were used for ELISA. In detail TNF-alpha concentration was titrated by sandwich ELISA, using the TNF-alpha Duo-Set kit (R&D, distributed by DBA Italia, Milan, Italy), essentially according to the manufacturer instructions. Each hippocampus homogenate was diluted (1:10, 1:30, and 1:60) in the assay. Data were reported as pg of TNF-alpha per mg of proteins.

### Real-Time PCR Analysis

Total RNA was extracted according to the TRI-Reagent (Sigma–Aldrich) protocol. The concentration and purity of RNA samples were determined by UV absorbance spectrophotometry. RNA integrity was checked on 1.2 % agarose gel electrophoresis. First-strand cDNA was synthesized from each total RNA (1 μg) using the QuantiTect reverse transcription kit (Qiagen). The Real Time PCR reactions were carried out in quadruplicate in an Applied Biosystems 7500 Real Time System by using the Power SYBR Green Master Mix PCR (Applied Biosystems). Each SYBR Green reaction (20 μl total volume) contained 2 μl of 1:1 diluted cDNA as template. For internal standard control, the expression of β-actin gene was quantified. Primer sequences were designed using Primer Express software (Applied Biosystems). Hpt primers were designed on the exon junction 278/279 (forward primer) on template NM012582.2 (forward primer, 5′- TGAGGCAGTGTGTGGGAAGCCC-3′; reverse primer, 5′- TGTGGCCCCAGTGGTGAGTCC-3′); β-actin primers were designed on the exon junction 75/76 (forward primer) on template NM031144.2 (forward primer, 5′- ACCCGCCACCAGTTCGCCAT-3′; reverse primer, 5′- CGGCCCACGATGGAGGGGAA-3′). Amplicons were 128–136 bp long. The PCR was performed under the following conditions: holding stage of 95°C per 10′; cycling tage (45 cycles): 95°C × 10 s – 60°C × 10 s – 72°C × 10 s; melting stage: 95°C × 5 s – 65°C × 1 m – 95°C × 30 s – 40°C × 30 s.

PCR amplification efficiencies were determined and ΔΔCT relative quantification was done using β-actin gene expression as internal control to normalize the results.

### Electrophoresis and Western Blotting of Hippocampal Homogenates

Proteins were extracted from hippocampus by homogenizing frozen tissues (-80°C) in ten volumes (w/v) of cold RIPA buffer (150 mM NaCl, 50 mM Tris-HCl, 1% NP-40, 0.5% sodium deoxycholate, 0.1% SDS, pH 8) containing Tissue Protease Inhibitor Cocktail (Sigma–Aldrich, 1:1000, v/v). Homogenates were then centrifuged (14,000 *g*, 45 min, 4°C) and protein concentration of supernatants was measured according to a published procedure (Bradford, 1976).

Aliquots (50 μg) of each homogenate were fractionated by electrophoresis in denaturing and reducing condition on 12.5% polyacrylamide gel, as previously reported (Spagnuolo et al., 2014a). After electrophoresis, proteins were transferred onto PVDF membrane (1 h, under electric field), the membrane was rinsed in T-TBS and then blocked with T-TBS containing 5% non-fat milk (overnight, 4°C). The membrane was then incubated (1 h, 37°C) with rabbit anti-rat Hpt (1: 500 dilution in T-TBS containing 0.25% non-fat milk) followed by GAR-HRP IgG (1: 500 dilution; 1 h, 37°C). The immunocomplexes were detected by the ECL detection system, using luminol as substrate, according to the manufacturer protocol. Quantitative densitometry of Hpt was then carried out by analyzing the digital images of membranes by the Gel-Pro Analyzer software (Media Cybernetics, Silver Spring, MA, USA). Band intensities were calculated as integrated optical density (IOD).

After Hpt detection, the membrane was extensively washed with T-TBS and then submerged in stripping buffer (100 mM β-mercaptoethanol, 2% SDS, 62.5 mM Tris-HCl, pH 6.7; 45 min, 50°C) for reprobing with anti-β-actin. After washing with T-TBS, the membrane was incubated (1 h, 37°C) with mouse anti-β-actin IgG (1:2,000 dilution), followed by GAM-HRP IgG (1:10,000 dilution; 1 h, 37°C). The immunocomplexes were detected by the ECL detection system, and densitometric analysis of the signal was carried out as above. Hpt concentration was then quantified after normalization with β-actin, and results were expressed as arbitrary units.

### Analysis of Hb Concentration in Rat Hippocampus

Haemoglobin concentration in individual samples was measured by ELISA. Samples were diluted (1:100, 1:400, 1:600, and 1:1000) with coating buffer, and then incubated in the wells of a microtiter plate (overnight, 4°C). After four washes by T-TBS and four washes by high salt TBS, the wells were blocked as above described (1 h, 37°C). After washing, the wells were incubated (1 h, 37°C) with 60 μl of rabbit anti-human Hb IgG (1: 500 dilution in T-TBS containing 0.25% BSA) followed by 60 μl of GAR-HRP IgG (1:5000 dilution). Peroxidase-catalyzed color development from *o*-phenylenediamine was measured at 492 nm. The calibration curve was obtained by assaying the immunoreactivity of 1, 0.5, 0.25, 0.1, and 0.05 ng of commercial Hb standard. Data were reported as μg of Hb per mg of proteins.

### Titration of Nitro-Tyrosine (N-Tyr) in Rat Hippocampus

N-Tyr in individual samples was quantified by ELISA. Samples were diluted (1:100, 1:250, 1:500, and 1:1000) with coating buffer, and aliquots (50 μl) were then incubated in the wells of a microtiter plate (overnight, 4°C). After four washes by T-TBS and four washes by high salt TBS the wells were blocked with TBS containing 0.5% BSA (1 h, 37°C). After washing, the wells were incubated (1 h, 37°C) with 50 μl of rabbit anti-N-Tyr (1: 1000 dilution in T-TBS containing 0.25% BSA) followed by 60 μl of GAR-HRP IgG (1:5000 dilution). Peroxidase-catalyzed color development from *o*-phenylenediamine was measured at 492 nm. Data were reported as OD per mg of proteins.

### Hpt Secretion by U-87 MG Cell Line

U-87 MG were seeded into six well-plate (400,000 cells/well) in complete medium and cultured for 20 h. After medium removal, cells were rinsed with DMEM, and incubated (20 h, 37°C) in DMEM containing different amounts of free cholesterol (0, 1.5, 3, 6, or 12 μg/ml), or liposome embedded 2-linoleoyl-1-palmitoyl-sn-glycero-3-phosphocholine (Lipo-LP; 0, 0.1, 0.3, 0.9, or 3 μM), or liposome-embedded PC (Lipo-PC; 0, 0.3, 0.9, 1.6, or 3 μM). At the end of incubation, media samples were collected, supplemented with Tissue Protease Inhibitor Cocktail (Sigma–Aldrich, 1:500, v/v), cleared of any cellular debris by centrifugation (400 *g*, 10 min), and finally immunoprecipitated with 1 μg of rabbit anti-human Hpt (overnight, 4°C). Twenty microliter of protein G Plus/protein A Agarose Suspension were added to each sample, and a further incubation (2 h, 4°C) was carried out.

The cells were extensively washed with DMEM, detached by treatment (5 min, 37°C) with 500 μl of trypsin (TrypLE Express, Gibco), and finally lysed with 0.3 mL of RIPA buffer (150 mM NaCl, 50 mM Tris-HCl, 0.5% NP-40, 0.5% sodium deoxycholate, 0.1% SDS, pH 8) containing the protease inhibitor cocktail (1:200, v/v). The lysates were centrifuged (12000 *g*, 30 min, 4°C) and then analyzed for their protein concentration (Bradford, 1976).

Immunoprecipitates were analyzed by 12.5% SDS-PAGE, and Western blotting for revealing Hpt. In detail, after protein transfer onto PVDF membrane, the blocking was performed as above described, and Hpt was revealed by incubation (overnight, 4°C) with mouse anti-human Hpt IgG (1: 500 dilution), followed by GAM-HRP IgG (1: 8000 dilution; 1 h, 37°C) and ECL staining. Quantitative densitometry of the bands was carried out by analyzing digital images of X-ray films exposed to immunostained membranes. In particular, the images were analyzed by the Gel-Pro Analyzer software (Media Cybernetics, Silver Spring, MA, USA), and the intensities of the bands were recorded as peaks on densitograms. The peak areas were measured and expressed as IOD, namely IOD. Band intensities (IOD) per mg of cell protein were then calculated.

Liposomes containing PC or LP were prepared by the cholate dialysis procedure as previously reported (Chen and Albers, 1982).

### Cell Viability Assay

In order to evaluate the effect of cholesterol, Lipo-LP, or Lipo-PC on survival of U-87 MG, cells were seeded into 96-well plate (8,000 cells per well), as above described, and cultured for 20 h in complete medium. After medium removal, cells were rinsed with DMEM, and incubated (20 h, 37°C) in DMEM containing different amounts of free cholesterol (0, 1.5, 3, 6, 12, 24, or 40 μg/ml), or Lipo-LP (; 0, 0.1, 0.3, 0.9, 3, 6, or 12 μM), Lipo-PC (0, 0.3, 0.9, 1.6, 3, 6, or 12 μM). The medium was then removed and cell survival was evaluated by MTT reduction assay. In detail, 100 μl of MTT (0.5 mg/ml in DMEM without Phenol red) were added to each well, and the incubation was carried out for 3 h at 37°C. One hundred microliters of a solution containing 0.1 M HCl in isopropanol were then added to each well, and, after 30 min, the absorbance at 595 nm was measured with a Bio-Rad 3350 microplate reader. The data were expressed as viability percentage, assuming the absorbance value from untreated cells as 100%.

### Statistical Analysis

In all experiments samples were processed in triplicate, and data were expressed as mean value ± SEM. The program “GraphPad Prism 5.01” (GraphPad Software, San Diego, CA, USA) was used to perform regression analysis, Student’s *t*-test, for comparing two groups of data, and one-way ANOVA, followed by Tukey’s test, for multiple group comparisons. *P* < 0.05 was set as indicating significance.

## Results

### General Features of High Fat Diet Fed Rats

As shown in **Table 2**, animals fed HFD showed a significantly higher body weight compared to control groups, both after 12 (574.0 ± 16.6 g vs. 640.0 ± 22.2 g, *p* = 0.045) and 24 weeks (614.3 ± 13.1 g vs. 714.5 ± 20.1 g, *p* = 0.045). In addition, weight gain was higher in the HFD group compared to CD group both after 12 (296.2 ± 21.6 g vs. 231.5 ± 15.8 g; 86 ± 6.2% vs. 66.1 ± 4.7%; *p* = 0.04) and 24 weeks (372 ± 20.6 g vs. 269 ± 11.8 g; 108.6 ± 6.6% vs. 77.7 ± 3.15%; *p* = 0.0004). The diet-dependent increase in body weight was also affected by the duration of diet administration (*p* < 0.05), in the HFD group (*p* = 0.03), while was not affected by the length of treatment in the control group (**Table 2**). Further, the analysis of body composition after 12 weeks of treatment revealed that lipid content was higher in HFD than in CD rats (33.06 ± 1.13% vs. 15.18 ± 0.38%; *p* < 0.001). Water and protein contents were significantly lower in HFD rats compared to CD rats (water, 48.46 ± 1.08% vs. 63.22 ± 0.44%, *p* < 0.001; protein, 12.82 ± 0.40% vs. 14.40 ± 0.46%, *p* = 0.03).

**Table 2:** Body weight and serum parameters.

	Control		High fat diet	
		
	12 weeks	24 weeks	12 weeks	24 weeks
Body weight (g)	574.0 ± 16.6^a^	614.3 ± 13.1^a^	640.0 ± 22.2^b^	714.5 ± 20.1^c^
Weight gain %	66.11 ± 4.70^a^	78.00 ± 2.90^a^	86.12 ± 6.20^b^	108.61 ± 6.61^c^
Cholesterol (mg/dL)	65.17 ± 0.75^a^	66.00 ± 1.82^a^	75.00 ± 0.92^b^	77.40 ± 0.68^b^
Triglycerides (mg/dL)	103.7 ± 3.43^a^	118.4 ± 4.08^b^	128.5 ± 4.90^b^	139.6 ± 2.06^c^
ALT (U/I)	66.67 ± 2.01^a^	72.80 ± 1.36^b^	79.83 ± 1.30^c^	91.00 ± 1.78^d^
Hepatic Triglycerides (mg/g)	5.10 ± 0.10^a^	6.40 ± 0.20^b^	10.20 ± 0.30^c^	13.70 ± 0.20^d^


*ALT, alanine amino transferase. Data are the means ± SE. Data with different superscripted letters are significantly different (*p* < 0.05).*

Cholesterol serum level was significantly higher in rats receiving HFD for 12 or 24 weeks compared to those receiving the CD for the same period (*p* < 0.001), but it was not affected by the duration of treatment. Serum and hepatic triglycerides levels were higher in HFD fed rats than in CD rats, in both groups (*p* < 0.001). In addition, a time-dependent effect was observed, as triglycerides concentration was more elevated in rats fed for 24 weeks compared to those fed for 12 weeks, in both CD and HFD groups (*p* < 0.001). A similar result was obtained by evaluating serum ALT (**Table 2**) as marker of hepatic function. Indeed ALT was higher (1.18 ± 0.01-fold, *p* < 0.001) in rats receiving HFD for 12 or 24 weeks respect to those receiving CD for the same period, as well as in 24 weeks fed rats compared to 12 weeks in both CD and HFD groups.

### Serum Level of Markers of Inflammation

**FIGURE 1:**
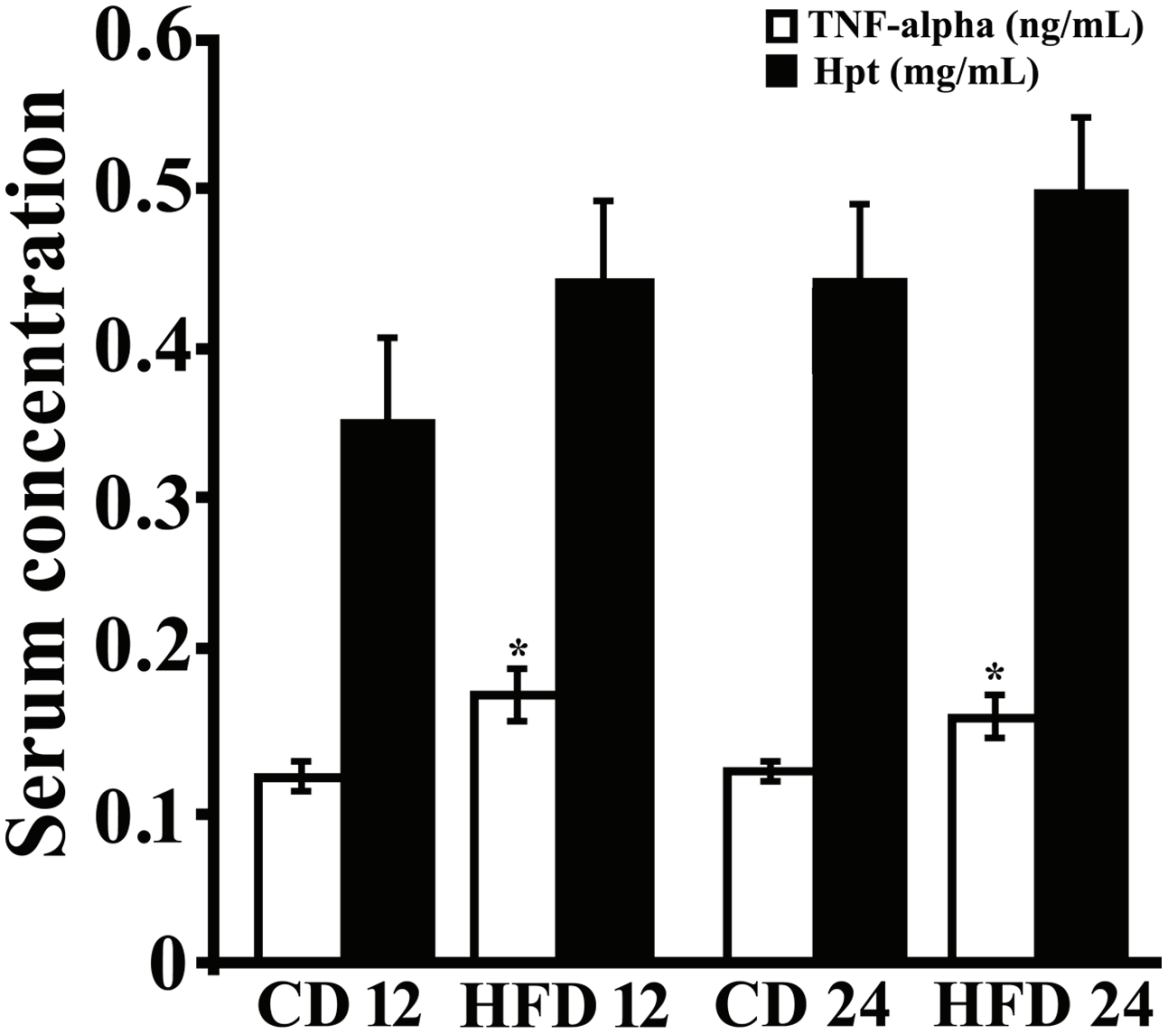
**Serum level of inflammation markers.** TNF-alpha (open bar) and Haptoglobin (Hpt; black bar) concentrations were measured by ELISA in serum of rats fed high-fat diet (HFD) or standard diet (control diet, CD) for 12 (CD 12, *N* = 8; HFD 12, *N* = 6) or 24 (CD 24, *N* = 7; HFD 24, *N* = 7) weeks. Each sample was analyzed in triplicate. Data were expressed as ng/ml (TNF-alpha) or mg/ml (Hpt), and reported as mean ± SEM. Significant differences between groups are indicated (^∗^, values significantly different from control by Student’s *t*-test, *p* < 0.05).

In order to study the effect of HFD diet on systemic markers of inflammation, serum TNF-alpha and Hpt levels were measured (**Figure 1**). TNF-alpha concentration was significantly higher (*p* < 0.05) in rats receiving HFD for 12 or 24 weeks respect to those receiving CD for the same period. TNF-alpha was found not to be time-dependent in CD group (CD 12, 0.122 ± 0.014 ng/mL; CD 24, 0.125 ± 0.006 ng/mL) or in HFD group (0.161 ± 0.02 ng/mL; HFD 24, 0.158 ± 0.013 ng/mL).

Serum Hpt level did not differ between control and treated rats both at 12 and 24 weeks. A time-dependent trend of increase in Hpt concentration was observed (CD 12, 0.353 ± 0.082 mg/mL; CD 24,0. 0.447 ± 0.031 mg/mL; HFD 12, 0.441 ± 0.088 mg/mL, HFD 24, 0.503 ± 0.038 mg/mL), although this was not significant in all groups.

It was previously demonstrated that in obese mice, which show increased serum Hpt after 12 weeks of treatment, Hpt expression was increased in WAT, but not in liver, suggesting that obesity induces a specific WAT Hpt upregulation (Lisi et al., 2011). In our experimental conditions, HFD fed rats showed a Hpt concentration 1.2-fold higher than CD rats, but this difference was not significant (0.441 ± 0.088 mg/mL vs. 0.353 ± 0.082 mg/mL). Conversely, serum Hpt, after 4 weeks of diet administration, was significantly higher in HFD than in CD rats (0.295 ± 0.022 mg/mL vs. 0.185 ± 0.019 mg/mL, *p* = 0.03; data not shown). This led us to suppose that in our experimental model (rat fed a diet rich in lard) a significant increase of Hpt just occurs in the initial phase of treatment.

### TNF-Alpha Concentration in Rat Hippocampus

**FIGURE 2:**
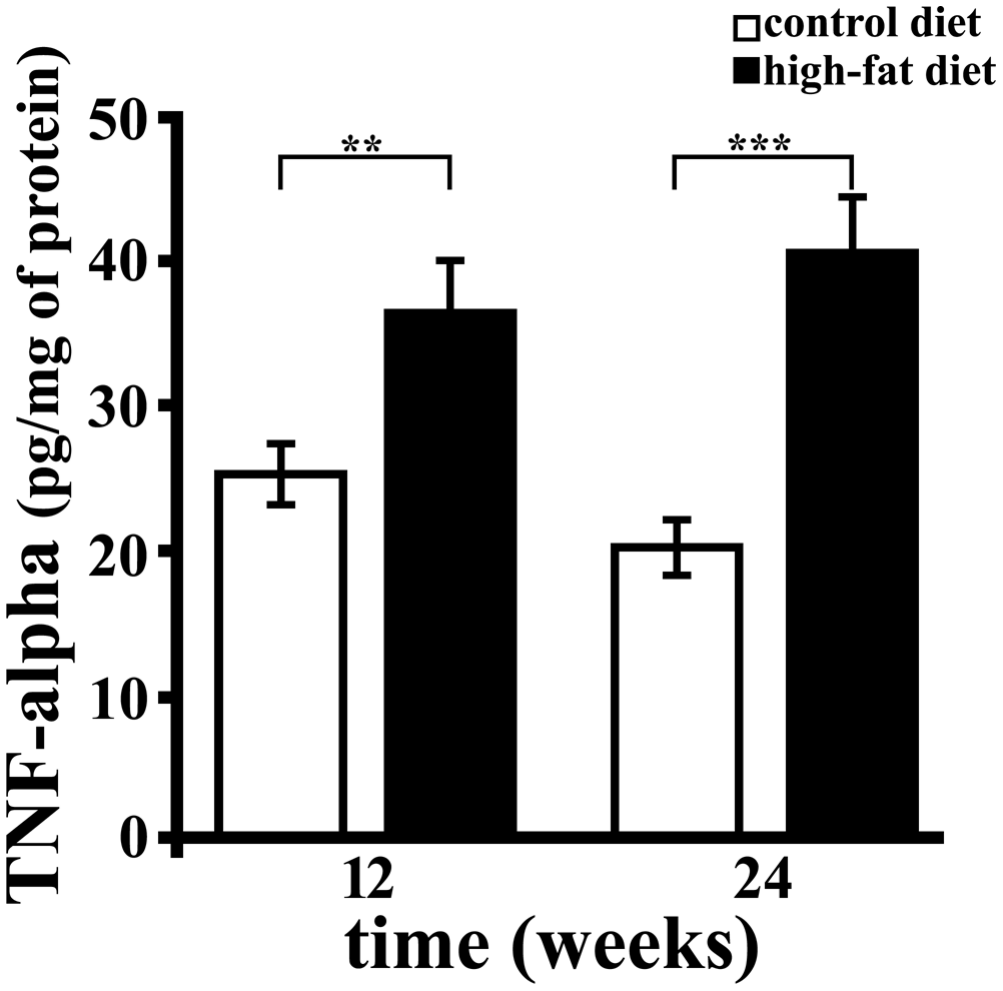
**TNF-alpha concentration in rat hippocampus.** TNF-alpha was titrated by sandwich ELISA in hippocampus of rats fed high-fat diet (HFD, black bar) or standard diet (control diet, CD, open bar) for 12 or 24 weeks. Each sample was analyzed in triplicate. Data were expressed as pg per mg of protein, and are reported as mean ± SEM. Significant differences between groups are indicated (^∗∗^values significantly different from control by Student’s *t*-test, *p* < 0.01; ^∗∗∗^values significantly different from control by Student’s *t*-test, *p* < 0.001).

TNF-alpha was titrated by sandwich ELISA, and significant diet-related changes of its level in hippocampus were observed (**Figure 2**). In particular, TNF-alpha concentration was about 1.5 (*p* < 0.01) or twofold (*p* < 0.001) higher in rats receiving HFD for 12 or 24 weeks compared to those receiving the CD for the same period. Further, we found that the duration of HFD administration did not affect hippocampal concentration of this cytokine. These results demonstrate that HFD, containing mainly lard as source of fat, induces brain inflammation, in agreement with previously published data (Thirumangalakudi et al., 2008; Wang et al., 2012), and that the effect was detectable within 12 weeks.

### Analysis of Hpt in Rat Hippocampus

**FIGURE 3:**
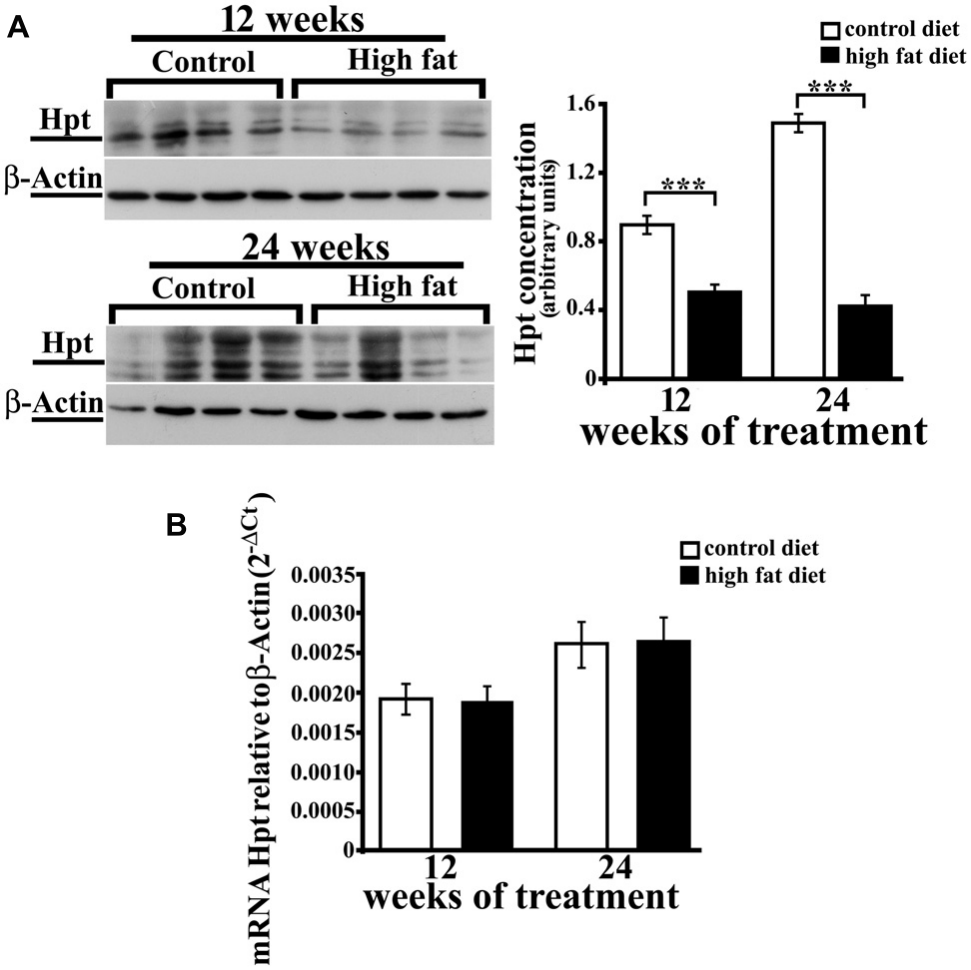
**Hpt expression in rat hippocampus.** Haptoglobin protein **(A)** and mRNA **(B)** levels in hippocampus of rats fed high-fat (HFD) or standard diet (control diet, CD) for 12 or 24 weeks. **(A)** On the left, representative Western blots are shown. Samples were analyzed by 12.5% SDS-PAGE and western blotting. Immunocomplexes were detected by rabbit anti-Hpt and GAR-HRP IgGs. After Hpt detection, the membrane was stripped and reprobed with anti-β-actin. On the right, quantitative densitometry of Hpt and β-actin was carried out and band intensities were calculated. Hpt concentrations are shown relative to β-actin level. Significance of differences is shown. ^∗∗∗^values significantly different from control by Student’s *t*-test, *p* < 0.001. **(B)** mRNA level of Hpt is shown relative to the β-actin mRNA. The data represent the mean ± SEM.

We previously reported that aging is associated with an increase of Hpt concentration and mRNA level in rat hippocampus (Spagnuolo et al., 2014a). As no data are available on the effect of HFD on brain expression of Hpt, we evaluated Hpt mRNA and protein levels in hippocampus of rats fed a HFD for 12 or 24 weeks. Hpt concentration, as detected by Western blotting, was significantly affected by the diet. As a matter of fact, Hpt level was lower (*p* < 0.001, **Figure 3A**) in hippocampus of HFD rats than in control animals, both in the group receiving the diet for 12 weeks (HFD 12, about 1.9-fold) and in 24 weeks group (HFD 24; about 3.5-fold). Further, in control animals, we found that Hpt concentration was about twofold higher (*p* < 0.001) in hippocampus of CD 24 compared with CD 12 rats, in line with our previously published data (Spagnuolo et al., 2014a), while the duration of HFD had no effect on Hpt concentration. Conversely, Real Time-PCR revealed no significant diet-related changes of Hpt expression (**Figure 3B**). An *in silico* analysis was performed using the RegRNA2.0 platform (http://regrna2.mbc.nctu.edu.tw/; Chang et al., 2013), and no AU-rich regions (ARE) in Hpt mRNA were found, thus suggesting that the observed diet-dependent decrease of protein concentration might be due to diet effects on Hpt synthesis and/or on Hpt stability.

### Analysis of Hb and N-Tyr Concentration in Hippocampus

**FIGURE 4:**
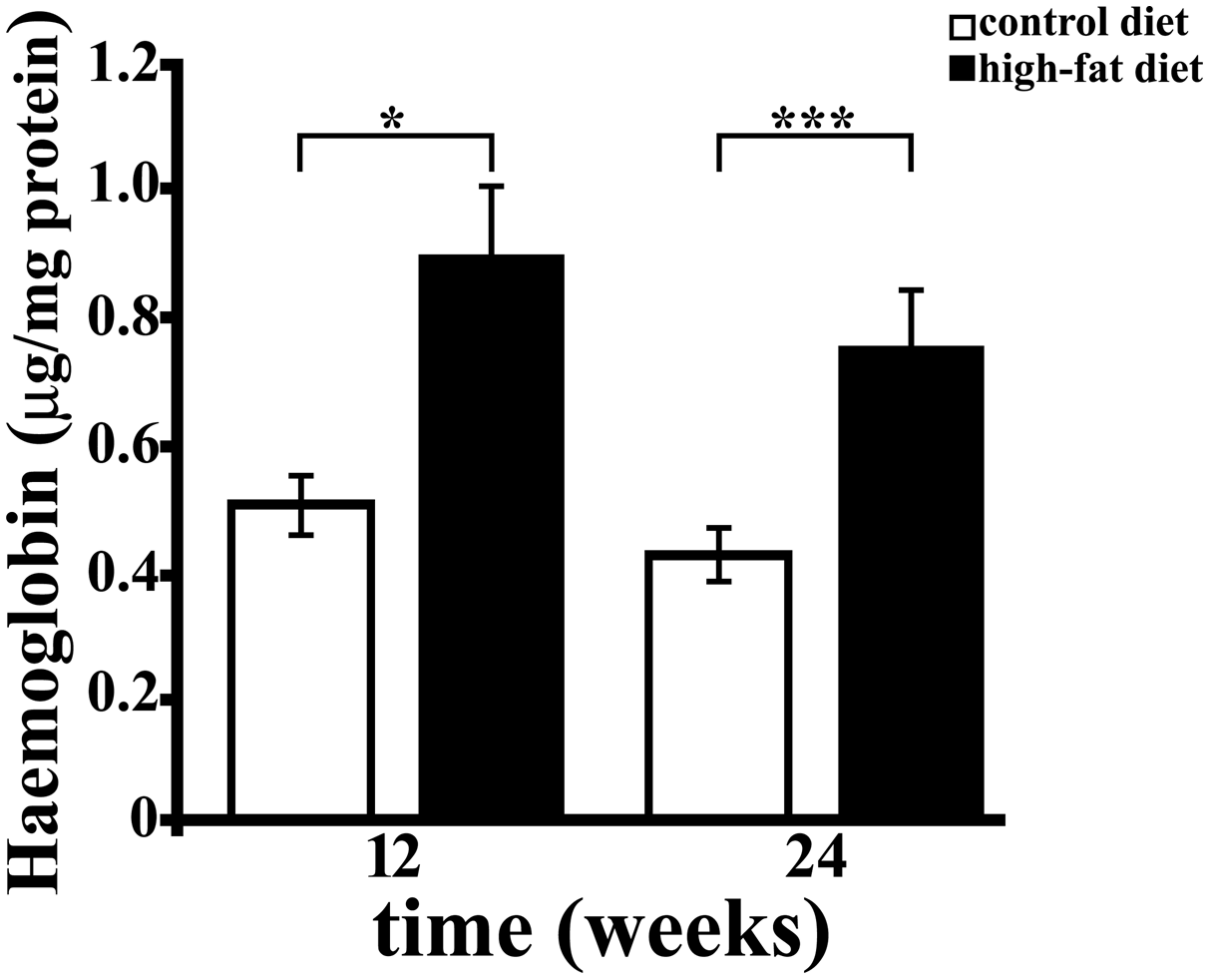
**Haemoglobin (Hb) concentration in rat hippocampus.** Hb level was measured by ELISA in hippocampus of rats fed HFD or control diet (CD) for 12 or 24 weeks. Each sample was analyzed in triplicate. Data were expressed as μg per mg of protein, and are reported as mean ± SEM. Significant differences between groups are indicated (^∗^values significantly different from control by Student’s *t*-test, *p* < 0.05; ^∗∗∗^values significantly different from control by Student’s *t*-test, *p* < 0.001).

Free Hb is highly toxic (Alayash, 2004), due to the presence of heme, that disrupts the lipid bilayers, and to the well known ability of iron to cause protein oxidation *in vivo* and *in vitro* (Qu et al., 2005). Hb was also reported to induce reactive oxygen and nitrogen species (superoxide radical and nitric oxide) production (Katsu et al., 2010; Yang et al., 2013). We therefore investigated whether HFD affects brain Hb concentration as well. Hb level, as assessed by ELISA, was significantly higher (about 1.8-fold, *p* < 0.01) in hippocampus of rats receiving the HFD for 12 or 24 weeks than in rats receiving the CD, while it was not affected by the duration of diet (**Figure 4**). Although a trend toward a time-dependent decrease in concentration was observed, the duration of treatment did not significantly affect hippocampal level of Hb neither in CD (CD 12, 0.489 ± 0.056 μg/mg protein; CD 24, 0.427 ± 0.033 μg/mg protein) nor in HFD group (HFD 12, 0.806 ± 0.114 μg/mg protein, HFD 24, 0.763 ± 0.072 μg/mg protein).

**FIGURE 5:**
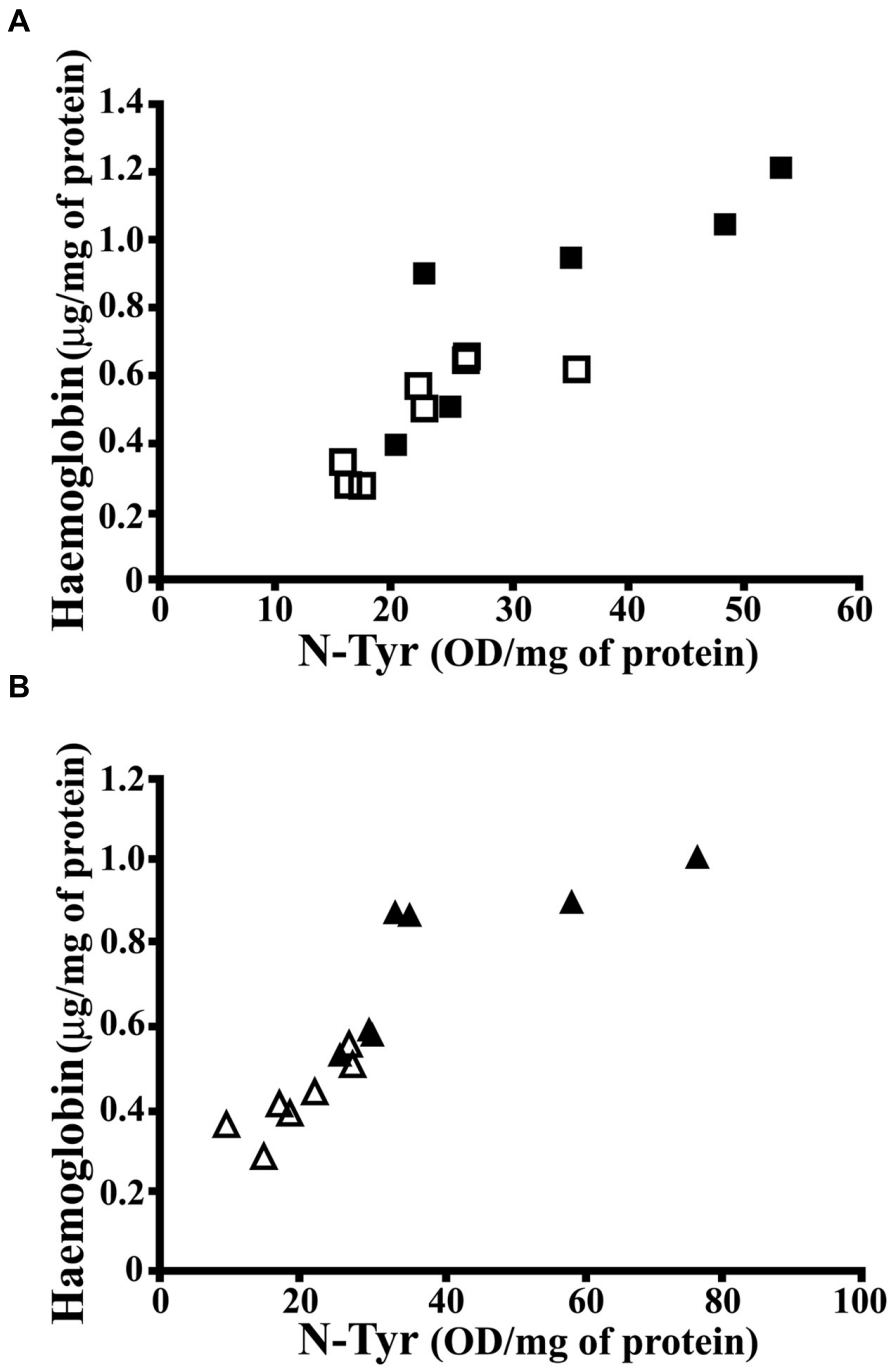
**Correlation between N-Tyr and Hb concentration in rat hippocampus.** **(A)** Hb and N-Tyr concentrations were measured by ELISA in hippocampus of rats fed high-fat (HFD, full squares) or control (CD, open squares) diet for 12 weeks. The statistical program Graph Pad Prism 5.01 performed the regression analysis and the calculation of P (CD, *r* = 0.815, *p* = 0.014; HFD, *r* = 0.837, *p* = 0.038). **(B)** Hb and N-Tyr concentrations were measured by ELISA in hippocampus of rats fed high-fat (HFD, full triangles) or control (CD, open triangles) diet for 24 weeks. The statistical program Graph Pad Prism 5.01 performed the regression analysis and the calculation of P (CD, *r* = 0.855, *p* = 0.014; HFD, *r* = 0.786, *p* = 0.036).

As known, Hpt binds with high affinity free Hb, thus preventing Hb-related oxidative damage and also limiting the release of heme (Quaye, 2008). The finding of higher level of Hb in hippocampus of HFD fed rats, with lower Hpt concentration, led us to hypothesize that the diet-dependent decrease of Hpt, which is known to act as antioxidant, might be associated with a higher extent of oxidative stress. In order to verify this hypothesis, we analyzed N-Tyr level in hippocampus of CD and HFD fed rats as marker of oxidative stress. It was previously reported that HFD administration to rats with a maternal history of HFD consumption prior to pregnancy is associated with the increase of N-Tyr level in cerebral cortex (White et al., 2009). We found that N-Tyr level was more elevated in hippocampus of HFD than in CD rats in both 12 weeks (35.04 ± 5.289 vs. 22.95 ± 2.318 OD/mg protein, *p* = 0.04) and 24 weeks groups (41.10 ± 7.163 vs. 19.62 ± 2.355 OD/mg protein, *p* = 0.01). These results support the hypothesis that HFD might induce protein oxidative damage in brain. Further, a positive correlation between Hb and N-Tyr concentration was found in each group (**Figure 5**; CD 12, *r* = 0.815, *p* = 0.014; HFD 12, *r* = 0.837, *p* = 0.038; CD 24, *r* = 0.855, *p* = 0.014; HFD 24, *r* = 0.786, *p* = 0.036), in line with recent findings demonstrating that Hb induces peroxynitrite formation *in vivo* (Ding et al., 2014, 2015). These data suggest that HFD is accompanied by an increase of Hb level in hippocampus which, in turn, is associated with protein oxidative modification.

### Cholesterol and Phospholipids Effect on Hpt Production by U-87 MG Astrocytes

We previously demonstrated that U-87 MG astrocytes are able to synthesize and secrete Hpt (Maresca et al., 2015). As HFD was reported to alter brain lipid metabolism and composition (Freeman et al., 2014) and our results show an effect on Hpt brain level, we investigated whether Hpt production by human astrocytes can be influenced by lipid treatment. To this aim, we choose for the *in vitro* treatment, cholesterol, LP, and PC that in terms of fatty acids composition are similar to lard, the major component of HFD used for rats treatment. Preliminarily, the effect of these compounds on U-87 viability was evaluated by MTT assay, in order to choose lipid concentration not affecting cell survival (data not shown).

**FIGURE 6:**
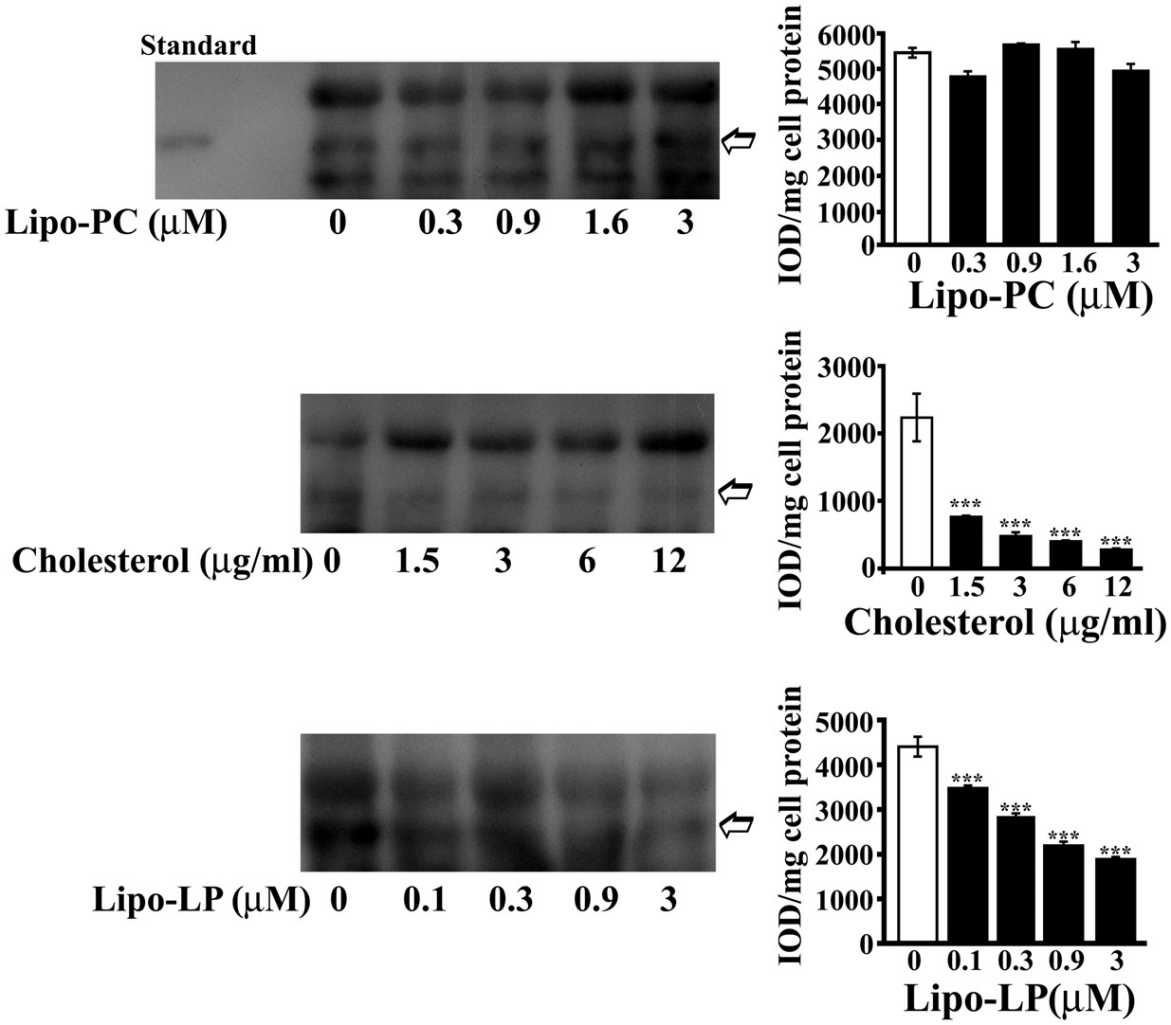
**Haptoglobin secretion by U-87 MG.** U-87 MG were incubated into six well-plate (20 h; 400,000 cells/well) in DMEM containing different amounts of liposome-embedded PC (Lipo-PC; 0, 0.3, 0.9, 1.6, or 3 μM), or free cholesterol (0, 1.5, 3, 6, or 12 μg/ml), or liposome embedded LP (Lipo-LP; 0, 0.1, 0.3, 0.9, or 3 μM). At the end of incubation, media samples were collected, immunoprecipitated with rabbit anti-human Hpt, and finally analyzed by electrophoresis on 12.5% polyacrylamide gel, under denaturing and reducing conditions, and Western Blotting. Representative Western blotting are shown **(left)**. Hpt in the supernatants (indicated with an arrow) was detected by mouse anti-human Hpt IgG and GAM-HRP IgG. Standard, commercial Hpt 1-1 (50 ng). Quantitative densitometry was carried out by the Gel-Pro Analyzer software **(right)**. The intensities of the bands were measured and expressed as IOD, namely Integrated Optical Density. Band intensities (IOD) per mg of cell protein were then calculated. Significance of difference from control (cells cultured in the absence of treatment; open bar) is shown. ^∗∗∗^*p* < 0.001.

U-87 were incubated (20 h, 37°C) in DMEM containing different amounts of Lipo-PC (0, 0.3, 0.9, 1.6, or 3 μM), or free cholesterol (0, 1.5, 3, 6, or 12 μg/ml), or Lipo-LP (0; 0.1, 0.3, 0.9, or 3 μM). After each treatment, cell culture supernatants were immunoprecipitated by monoclonal anti-human Hpt and then immunostained with polyclonal anti-Hpt. A significant dose-dependent decrease of Hpt level in supernatants from astrocyte treated with cholesterol or Lipo-LP was found. In particular, the amount of Hpt was reduced of about threefold by treatment with 1.5 μg/ml cholesterol (*p* < 0.001) and of about fourfold by treatment with 3 μg/ml cholesterol (*p* < 0.001). As shown in **Figure 6**, a sevenfold decrease (*p* < 0.001) of Hpt amount was induced by cell incubation with 12 μg/ml cholesterol. Lipo-LP treatment also impaired Hpt secretion, inducing a 2.5-fold decrease of Hpt level (*p* < 0.001) at the higher concentration used. Conversely, Lipo-PC treatment did not affect Hpt secretion. These results suggest that cholesterol and fatty acids, such as palmitic and linoleic acid, can interfere with Hpt release by U-87 MG in the extracellular compartment.

## Discussion

The neurodegeneration and the inflammation/obesity are becoming an inseparable binomial association. Nutrition-related effects on brain function have been extensively investigated in the last decade, and it was strongly suggested that obesity and increased consumption of HFD increase risk for development of dementia (Luchsinger et al., 2002; Hebert et al., 2003; Solfrizzi et al., 2003; Craft, 2009), although it remains to clarify the molecular mechanisms responsible for these phenomena. Oxidative stress and inflammation were suggested to play a central role in causing HFD-induced damage to the brain (Freeman et al., 2014).

To unveil the biochemical and molecular mechanisms underlying the brain consequences of dietary fats, it could be useful to identify factors that significantly change in the brain following HFD administration. Hpt represents an interesting tool to further explore the relationship between diet and inflammation in brain. Beneficial effects of this protein were already shown, since it plays a critical role in brain, defending neurons from Hb-induced damage after intracerebral hemorrhage (Zhao et al., 2009). Also, Hpt prevents ApoE and ApoA-I oxidation (Salvatore et al., 2007, 2009), and modulates ApoE-mediated cholesterol trafficking in neuroblastoma cell lines (Spagnuolo et al., 2014a).

Here we demonstrate, for the first time, that Hpt protein level is significantly reduced in hippocampus of rats fed for a long term (until 12 or 24 weeks) a high fat diet. In contrast, Hpt mRNA did not change in response to dietary intervention. As no ARE regions, which are known to affect mRNA stability, were found in rat Hpt mRNA, differences between Real time and Western blotting results might be due to diet effects on Hpt synthesis and/or on Hpt stability. This discrepancy between mRNA and protein levels is observed for many proteins in mammalian cells, and may indicate that the modulation of HFD in the brain might be exerted mainly at the protein level (Vogel and Marcotte, 2012).

The concentration of TNF-alpha, a cytokine known to exert pleiotropic functions in central nervous system pathogenesis (McCoy and Tansey, 2008) was higher in hippocampus of high-fat fed rats, thus indicating a diet-induced inflammation, which might contribute to oxidative brain injury. Indeed it was reported that inflammation is associated with the release of reactive oxygen and nitrogen species (Urrutia et al., 2014), and with the activation of inducible nitric oxide synthase (iNOS) gene in astrocytes and microglia (Possel et al., 2000; Hewett and Hewett, 2012). In this study we detected a higher amount of Hb in hippocampus of HFD fed rats that exhibit lower Hpt content, compared to CD fed rats. It is worth mentioning that a quote of brain Hb can derive from the transient extravasation of erythrocytes or from neutrophils from the peripheral circulation (Chuang et al., 2012). As accumulating evidence suggest that HFD can alter vascular components of the brain, leading to BBB disruption and dysfunction of brain endothelial cells (Freeman et al., 2014), changes to vascularization/BBB integrity might be responsible for the observed increase of cerebral Hb. On the other hand, Hb was previously detected in neurons from rat brains (Richter et al., 2009; Schelshorn et al., 2009), and it was proposed it is responsible for O_2_ homeostasis in this area (Biagioli et al., 2009). Expression of Hb is enhanced by hypoxia (Schelshorn et al., 2009), and obesity-induced cerebral hypoperfusion, derived from endothelial dysfunction, was reported (Toda et al., 2014). Therefore, it cannot be excluded that the up-regulation of Hb that we observed might be a feedback response to hypoxia. Exogenous Hb released from red blood cells after traumatic brain injury or intracerebral hemorrhage is highly neurotoxic (Xi et al., 2006), and increased expression of Hpt was reported to protect neurons against hypoxic-ischemic injury (Zhao et al., 2009). As Hpt binds with extremely high affinity Hb, the reduction of brain Hpt might be associated with an increased brain damage. By forming Hpt-Hb complexes, Hpt stabilizes and shields heme iron within the hydrophobic pocket of Hb, thereby preventing its pro-oxidative properties and cytotoxicity (Yang et al., 2003; Quaye, 2008). Indeed, excessive Hb and iron overload induce oxidative injury and significantly contribute to brain damage (Xi et al., 2006). Further, it was previously reported that high fat diet induces oxidative stress and endothelial dysfunction in rats, essentially by down-regulation of antioxidant enzymes, such as superoxide dismutase, catalase, glutathione peroxidase, and heme oxygenase-2 (HO-2; Roberts et al., 2006). So it was expected a higher extent of oxidative damage in brain of HFD fed rats. We measured N-Tyr in rat hippocampus as marker of protein oxidation induced by peroxynitrite, which, in turn, is produced by reaction of superoxide and nitric oxide. Tyrosine nitration was reported to be involved in the pathogenesis of numerous neurodegenerative disorders (Sacksteder et al., 2006; Lee et al., 2009; Yeo et al., 2015), and increased protein tyrosine nitration in aorta, kidney, heart and liver of rats, in response to HFD administration, was previously described (Roberts et al., 2000; Ghosh et al., 2006; Bautista et al., 2014). As a matter of fact we found increased levels of N-Tyr in hippocampus of HFD fed rats, likely due to the higher level of Hb, which is known to induce superoxide radical, nitric oxide, and peroxynitrite production (Katsu et al., 2010; Yang et al., 2013; Ding et al., 2014, 2015). This pro-oxidative effect of Hb could be amplified by the reduction of Hpt, the major Hb-scavenger. Based on these data, we hypothesize that in the obese rat the metabolic activity of oligodendrocytes and astrocytes, which are the main source of Hpt in the brain (Zhao et al., 2009; Maresca et al., 2015), is altered by the HFD, and/or these cells are affected by oxidative stress and become less abundant in the brain population. In the attempt of unraveling this mechanism, we investigated the effect of cholesterol, LP or PC on Hpt synthesis by astrocytes *in vitro*. These molecules were chosen to mimic, also in terms of fatty acids composition, the effect of lard, which is the major component of the HFD used in this study. Our data demonstrate that Hpt secretion is significantly impaired by cholesterol or LP treatment, strongly suggesting that the release of this glycoprotein can be affected by the alteration of lipid composition of the brain.

## Conclusion

We report evidence that the administration of HFD is associated with the decrease of Hpt, and with the increase of Hb level and protein oxidative modification in hippocampus. These findings led us to hypothesize that Hpt decrease might enhance the oxidative stress induced by free Hb. In addition, as Hb was shown to promote the release of inflammatory cytokines by microglia (Wang et al., 2014), we speculate that Hpt, by binding Hb, might prevent this pro-inflammatory function. Therefore, the concomitant reduction of Hpt and increase of Hb level in brain of HFD rats might contribute to inflammatory cerebral injury. On the other hand, since Hpt actively participates in critical processes such as immune cells recruitment, tissue repair and regeneration (Quaye, 2008), it cannot be excluded that its reduction in brain may have important implications in diet enhanced brain damage. It remains to assess whether other cerebroprotective factors, such as HO-2, the O_2_ sensor in brain, that contributes to maintain neuronal physiological functions and to regulate the vascular tone of cerebral blood vessels (Parfenova and Leﬄer, 2008), are affected by diet administration. Nevertheless, our data, identifying Hpt as a molecule modulated in the brain by dietary fats, may represent one of the first steps in the comprehension of the molecular mechanism underlying the diet-related effects in the nervous system.

## Author Contributions

MSS, LC conceived and designed the research, performed cell biology and WB experiments, analyzed and interpreted all results, wrote the manuscript.

BM contributed to the planning of *in vitro* experiments, performed cell biology and WB experiments, analyzed data.

MPM, GC, CC, GT, MC contributed to the planning and execution of experiments with rats, analyzed data, and critically revised the manuscript.

RS contributed to the planning and execution of Real Time PCR experiments, analyzed results and critically revised the manuscript.

All authors approved the final version to be published, and ensured that questions related to the accuracy or integrity of any part of the work are appropriately investigated and resolved.

## Conflict of Interest Statement

The authors declare that the research was conducted in the absence of any commercial or financial relationships that could be construed as a potential conflict of interest.
